# Cortical diffusivity investigation in posterior cortical atrophy and typical Alzheimer’s disease

**DOI:** 10.1007/s00415-020-10109-w

**Published:** 2020-08-08

**Authors:** Mario Torso, Samrah Ahmed, Christopher Butler, Giovanna Zamboni, Mark Jenkinson, Steven Chance

**Affiliations:** 1 Nuffield Department of Clinical Neurosciences, University of Oxford, Oxford, UK; 2 Oxford Brain Diagnostics, Oxford Centre for Innovation, New Road, Oxford, OX1 1BY UK; 3 Research Institute for the Care of Older People, Royal United Hospital, Bath, UK; 4 Dipartimento di Scienze Biomediche, Metaboliche e Neuroscienze, Università di Modena e Reggio Emilia, Modena, Italy; 5 Wellcome Centre for Integrative Neuroimaging, FMRIB, Nuffield Department of Clinical Neurosciences, University of Oxford, Oxford, UK

**Keywords:** Cortical diffusivity, Diffusion tensor imaging, Posterior cortical atrophy, Typical Alzheimer’s disease, Minicolumn

## Abstract

**Objectives:**

To investigate the global cortical and regional quantitative features of cortical neural architecture in the brains of patients with posterior cortical atrophy (PCA) and typical Alzheimer’s disease (tAD) compared with elderly healthy controls (HC).

**Methods:**

A novel diffusion MRI method, that has been shown to correlate with minicolumnar organization changes in the cerebral cortex, was used as a surrogate of neuropathological changes in dementia. A cohort of 15 PCA patients, 23 tAD and 22 healthy elderly controls (HC) were enrolled to investigate the changes in cortical diffusivity among groups. For each subject, 3 T MRI T1-weighted images and diffusion tensor imaging (DTI) scans were analysed to extract novel cortical DTI derived measures (AngleR, PerpPD and ParlPD). Receiver operating characteristics (ROC) curve analysis and the area under the curve (AUC) were used to assess the group discrimination capability of the method.

**Results:**

The results showed that the global cortical DTI derived measures were able to detect differences, in both PCA and tAD patients compared to healthy controls. The AngleR was the best measure to discriminate HC from tAD (AUC = 0.922), while PerpPD was the best measure to discriminate HC from PCA (AUC = 0.961). Finally, the best global measure to differentiate the two patient groups was ParlPD (AUC = 0.771). The comparison between PCA and tAD patients revealed a different pattern of damage within the AD spectrum and the regional comparisons identified significant differences in key regions including parietal and temporal lobe cortical areas. The best AUCs were shown by PerpPD right lingual cortex (AUC = 0.856), PerpPD right superior parietal cortex (AUC = 0.842) and ParlPD right lateral occipital cortex (AUC = 0.826).

**Conclusions:**

Diagnostic group differences were found, suggesting that the new cortical DTI analysis method may be useful to investigate cortical changes in dementia, providing better characterization of neurodegeneration, and potentially aiding differential diagnosis and prognostic accuracy.

**Electronic supplementary material:**

The online version of this article (10.1007/s00415-020-10109-w) contains supplementary material, which is available to authorized users.

## Introduction

Posterior cortical atrophy (PCA) is typically an early onset neurodegenerative condition, characterised by progressive visuospatial and visuo-perceptual deficits, but relatively preserved memory [1–5].

For most patients, the underlying aetiology is Alzheimer’s disease (AD) [5, 6] so PCA is considered a rare variant, different from typical AD (tAD) [7–9]. However, other neurodegenerative processes sometimes underlie PCA [4–6] so PCA could be a distinct nosological entity [10, 11], frequently misclassified.

Better knowledge of the underlying pathology should improve diagnostic and prognostic accuracy and aid development of therapeutic strategies. Neuroimaging studies have shown different patterns of grey matter (GM) damage for PCA patients compared to controls or tAD, where the main differences involved occipito-temporal and parietal regions [11–17]. However, there is still poor knowledge about the microstructural alterations underlying these patterns of GM damage.

Diffusion tensor imaging (DTI) is a promising surrogate for the microstructural properties of brain tissue.

Although most such studies have focused on white matter (WM), in the last years, there is a growing interest in the detection of microstructural GM changes measuring the diffusion of water molecules in the cortex. Some interesting recent studies attempted to investigate the cortical changes in neurodegenerative conditions using the neurite orientation dispersion and density imaging method (NODDI) [18, 19]. This approach was designed to assess the cortical properties divided into three separate microstructural environments: neurites, extra-neurites, and cerebro-spinal fluid (CSF). However, this method requires a multi-shell acquisition, so it is not yet widely applied to dementia patient cohorts. It has been shown that NODDI metrics are significantly dependent on field strength [20].

The present study aimed to investigate cortical features in PCA, tAD and elderly healthy controls (HC), using DTI as a surrogate measure for cortical micro-anatomical alterations that are well known in neurodegenerative pathologies. A novel MRI analysis tool was applied, designed specifically for quantifying global cortical and regional DTI signals in GM related to cortical micro-geometry. The method has been tested using ex-vivo imaging comparisons with post-mortem histology, demonstrating that the DTI analysis method is sensitive to the minicolumnar cytoarchitecture in cortical GM [21]. The cortical minicolumn is a vertical string of neurons, with associated dendrites and myelinated axon bundles, which represents a fundamental component of the network architecture of the cerebral cortex [22–24] and its disruption has been identified as a neuropathological biomarker in dementia [25–27].

## Methods

### Participants

All subjects’ data in the study had been collected as part of previous studies. Fifteen PCA patients were recruited through the Oxford Cognitive Disorders Clinic, Oxford, UK. Diagnosis was established by a senior behavioural neurologist (CB) and neuropsychologist (SA). All patients fulfilled consensus criteria for PCA [4, 5], based upon clinical assessment, brain imaging and detailed neuropsychological assessment. Clinical magnetic resonance imaging (MRI) confirmed focal atrophy in the occipital and parietal lobes.

A tAD and a control group were used for comparisons with the PCA patients. Twenty-three tAD patients were recruited from the Oxford Project to Investigate Memory and Aging (OPTIMA) [28] and the Memory and Amnesia Project, University of Oxford, UK. All tAD subjects fulfilled consensus criteria for Alzheimer’s disease [29, 30], based upon clinical assessment, detailed neuropsychological assessment, and structural brain imaging.

Twenty-two comparable healthy controls were recruited in Oxford. Participants were recruited from the OPTIMA Project and from the Oxford Memory Assessment Clinic (OXMAC) at the John Radcliffe Hospital. Healthy volunteers were recruited as such if they did not have a subjective or reported memory complaint and performed within the normal range in global cognitive scales (i.e., MMSE > 26 and CDR = 0). These participants had no prior history of psychiatric illness, significant head injury, or cerebrovascular disease, and were not prescribed any medication known to affect cognition. Neuropsychological data were not comparable between groups due to the earlier acquisition of some data under different research protocols.

### MRI data acquisition protocol

Scanning for all subjects entered in the study was performed at the Oxford Centre for Clinical Magnetic Resonance Research using a 3 T Trio Siemens MRI scanner equipped with a 12-channel head coil. The neuroimaging protocol included: (1) diffusion weighted image (DWI) acquisition SE-EPI sequence, TR/TE = 9300/94 ms; resolution = 2 × 2 × 2 mm^3^; flip angle 90°; FOV = 192 mm; number of diffusion directions = 60; *b* value = 1000 s/mm^2^ and two additional images with no diffusion weighting (*b* = 0), (2) high-resolution T1-weighted 3D MP-RAGE images (TR/TE = 2040/4.7 ms; resolution = 1 × 1 × 1 mm^3^; FOV 192 mm).

### MRI preprocessing

The 3D T1-weighted image for each subject was segmented using FreeSurfer v 6.0 (https://surfer.nmr.mgh.harvard.edu/). This provided outputs containing estimates of the cortical grey matter volume, white matter (WM) volume, cortical surfaces (CS) and the cortical thickness (CT), based on the standard segmentation and surface-fitting performed by FreeSurfer.

DTI preprocessing was performed using FSL tools (FSL Version 6.0; FMRIB Software Library, Oxford, UK—https://www.fmrib.ox.ac.uk/fsl/). For each subject, the diffusion data were visually checked by two trained investigators (MT and SA) to detect artefacts and corrupted volumes. DTI scans of low quality were removed (1 AD and 1 PCA). Diffusion-weighted images were then corrected for motion and eddy current effects by alignment of all images to a reference *b* = 0 image using FSL’s eddy tool. The diffusion tensor was then calculated with the FSL DTIFIT tool, providing fractional anisotropy (FA), mean diffusivity (MD) and V1 maps. For each subject, the displacement among diffusion volumes was estimated using the eddy_correct output to obtain a measure of head movement (defined as “movement”) during the acquisition. To reduce the impact of partial volume effect, we used a 0.5 mm erosion from the edges. In previous testing, we explored four different degrees of erosion and 0.5 mm was selected as the best combination between reduction of partial volume effect and maintaining a sufficient number of voxels. The tests showed that any remaining partial volume effect due to contamination from CSF and WM, had little impact on the cortical diffusion measures.

### Cortical diffusivity analysis

Standard diffusivity analysis was conducted to calculate MD and FA in the cortex. Further cortical diffusivity analysis was performed using a proprietary software tool. The tool generates cortical profiles, i.e., radial lines within the cortex, providing an estimate of the columnar organisation within the cortex. Values for the diffusion tensor derived metrics were averaged along the cortical profiles, throughout cortical grey matter (method previously described) [21]. To summarise the method, three measures were calculated in this manner, relating to the principal diffusion component: the angle between the radial minicolumnar direction and the principal diffusion direction (AngleR, θrad); the principal diffusion component perpendicular to the radial minicolumnar direction [PerpPD, *D*1,⊥ (× 10^–3^ mm^2^/s)], and the principal diffusion component parallel with the radial minicolumnar direction [ParlPD, *D*1,∥ (× 10^–3^ mm^2^/s)] (see also the article by McKavanagh et al. [21] where AngleR was described as the difference between the radial minicolumn direction in the cortex and the principal diffusion vector, PerpPD and ParlPD were calculated by projecting the principal vector diffusion tensor component on to the planes perpendicular and parallel to the radial minicolumn direction in the cortex). All the cortical values were averaged across all of the cortical profiles within the region of interest (being the entire cortex for the whole brain value, or the cortical anatomical area for the regional values) to reduce the influence of noise in the DTI scans. This effectively smoothed the data, ensuring only directionality with some local coherence would dominate, guarding against the influence of random deflections from the radial direction. Previous work has found that measures of the cyto- and myelo-architecture are relatively stable within a cortical subregion [31] indicating that it is valid to find an average value for that region. Global cortical diffusion values included all regions of the cerebral cortex to generate overall values for the whole brain cerebral cortex. These were used to compare groups and the metrics that differentiated the three groups were then used for the regional analyses. To investigate the regional cortical diffusion metrics, single values for each cortical region were extracted using the regional segmentation provided by the recon-all pipeline of the FreeSurfer v 6.0 software package (https://surfer.nmr.mgh.harvard.edu/) based on Desikan-Atlas.

**Fig. 1:**
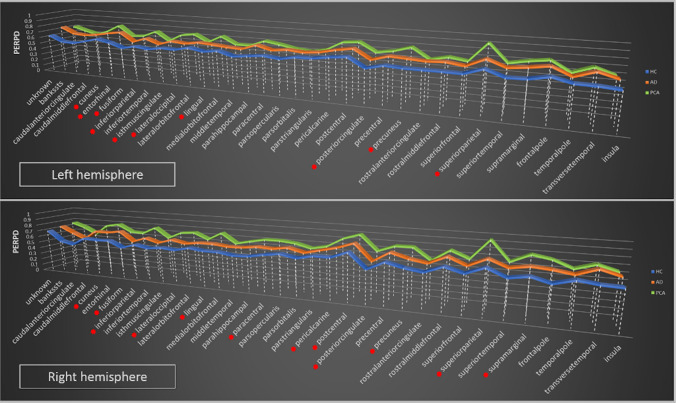
Regional PerpPD differences. It shows the group differences in regional PerpPD values. *Remained statistically significant after false discovery rate correction (FDR < 0.05; 210 tests)

### Volumetric assessment

The cortical GM mask, the WM mask and the CS mask obtained by Freesurfer recon-all were used to estimate the GM and WM volume. To account for head size, the GM, WM and CS volumes were normalised by the total intracranial volume (TIV) to obtain the GM fraction GM_fr,WM fraction (WM_fr) and CS fraction (CS_fr).

The right and left hippocampal volumes were automatically segmented using Freesurfer recon-all. Hippocampal volumes (left and right) were averaged and normalised by TIV to obtain the hippocampal fraction (Hipp_fr).

### Statistical analysis

Data were analysed using IBM SPSS Statistics version 25 (SPSS, Chicago, IL, USA). Group comparisons were performed using *t* tests or analysis of variance (ANOVA) for continuous variables, and chi-squared tests were used for dichotomous or categorical variables.

ANOVA was performed using the multivariate general linear model (GLM) in SPSS to compare the between-group differences in diffusion and volumetric measurements, using the diagnostic group as a fixed factor and the head movement and age as covariates. To investigate the associations between global cortical diffusivity, volume, and thickness, Partial Spearman’s rank correlations were used, accounting for age and gender.

All statistically significant results reported remained significant after false discovery rate correction (FDR < 0.05) [32]. Bonferroni correction was used to account for multiple comparisons in demographics (*p* < 0.05/9).

To investigate the discrimination power of the cortical diffusion measures (FA, MD, AngleR, PerpPD and ParlPD) and three conventional structural measures (GM_fr, CT and Hipp_fr), the Receiver operating characteristic (ROC) curves were used. Every measure entered into the ROC analysis was adjusted for age and head movement. The area under the curve was used as a diagnostic accuracy index.

## Results

### Participants

Table 1 summarises the principal demographic and clinical characteristics of all subjects entered in the study. No significant difference was observed between groups for years of formal education (*F*_2,55_ = 2.84; *p* = 0.07) or sex (Chi-square HC vs tAD = 0.0178; *p* = 0.89.; HC vs PCA = 0.1081; *p* = 0.74.; tAD vs PCA = 0.0451; *p* = 0.83). The PCA group was significantly younger than HC group (*F*_2,55_ = 9.47; *p* = 0.000). The illness duration was higher in the PCA group compared to tAD (*F*_2,36_ = 8.151; *p* = 0.048), but this was not significant after multiple comparisons correction. Age and movement were used as covariates in further GLM analysis.

**Table 1:** Demographics, volumetrics and head movement

	HC (*N* = 22)	tAD (*N* = 23)	PCA (*N* = 15)
Demographics			
Age (years)	74.7 (± 6.98)	74.9 (± 5.64)	65.4 (± 7.21)^#^
Gender M/F	12/10	13/10	9/6
Education (years)	15.57 (± 3.25)	13.36 (± 3.38)	13.7 (± 2.20)
Volumetrics			
Cortical GM_fr	0.319 (± 0.036)	0.265 (± 0.30)^#^	0.257 (± 0.024)^#^
WM_fr	0.165 (± 0.028)	0.151(± 0.203)	0.150 (± 0.012)
CS	0.1193 (± 0.144)	0.1075 (± 0.125)^#^	0.1071 (± 0.106)^#^
Cortical thickness	2.75 (± 0.10)	2.63 (± 0.15)^#^*	2.50 (± 0.15)^#^
Hipp_fr	0.00562 (± 0.00092)	0.00427 (± 0.00069)^#^*	0.00498 (± 0.00062)^#^
Head movement	0.698 (± 0.24)	0.742 (± 0.25)	0.700 (± 0.20)

The group differences for demographic and volumetric measures. Demographics measures were compared among groups using ANOVA. Bonferroni correction was used to account for multiple comparisons in demographics (*p* < 0.05/9). Volumetrics measures were compared using GLM multivariate. All *p* values in volumetrics remained statistically significant after false discovery rate correction (FDR < 0.05; 15 tests)

All values are expressed as: mean (standard deviation). ^#^statistically significant difference among patients (tAD or PCA) and HC. *Statistically significant difference among tAD and PCA

*HC* healthy controls, *tAD* typical Alzheimer’s disease, *PCA* posterior cortical atrophy, *Cortical GM_fr* cortical grey matter fraction, *WM_fr* white matter fraction, *CS* cortical surface, *Hipp_fr* hippocampal fraction

### Volumetric assessment

For global cortical volumetric measures, GLM analysis revealed a significant effect of diagnostic group on GM_fr (*F*_2,58_ = 32.679; *p* = 0.000), WM_fr (*F*_2,58_ = 6.222; *p* = 0.004), CT (*F*_2,58_ = 14.800; *p* = 0.000) and Hipp_fr (*F*_2,58_ = 18.539; *p* = 0.000). The between groups comparisons are summarized in Table 1. No significant effects of age or movement on volumetric variables were detected.

### Cortical diffusion measurements

#### Global

Table 2 summarises global cortical diffusivity results. GLM revealed significant effects of diagnostic group on AngleR (*F*_2,58_ = 18.932*; p* = 0.000), PerpPD (*F*_2,58_ = 27.348*; p* = 0.000), MD (*F*_2,58_ = 23.385*; p* = 0.000) and ParlPD (*F*_2,58_ = 23.144*; p* = 0.000). No significant differences between groups in FA were found. No significant effects of age or movement on diffusion values were detected. (FDR < 0.05; 5 tests).

**Table 2:** Global cortical diffusion measures

Global cortical DTI	HC (*N* = 22)	tAD (*N* = 23)	PCA (*N* = 15)
FA	0.1837 (± 0.007)	0.1785 (± 0.011)	0.1795 (± 0.012)
MD	1.0583 (± 0.053)	1.1688 (± 0.076)^#^	1.2341 (± 0.093)^#^
AngleR	0.9766 (± 0.0038)	0.9863 (± 0.0065)^#^	0.9853 (± 0.0059)
PerpPD	0.5932 (± 0.030)	0.6611 (± 0.049)^#^*	0.7085 (± 0.059)^#^
ParlPD	0.3963 (± 0.021)	0.4311 (± 0.030)^#^*	0.4650 (± 0.037)^#^

The group differences for each global cortical DTI measure considered in the study, investigated using t-tests. All values are expressed as: mean (standard deviation)

*HC* healthy controls, *tAD* typical Alzheimer’s disease, *PCA* posterior cortical atrophy, *FA* fractional anisotropy, *MD* mean diffusivity, *AngleR* angle between the radial minicolumnar direction and the principal diffusion direction, *PerpPD* the principal diffusion component perpendicular to the radial minicolumnar direction, *ParlPD* the principal diffusion component parallel with the radial minicolumnar direction

^#^Statistically significant difference among patients (tAD or PCA) and HC

^*^Statistically significant difference among tAD and PCA. Group differences were considered statistically significant after false discovery rate correction (FDR < 0.05; 15 tests)

The between groups are summarized in Table 2. Only PerpPD and ParlPD were significantly differentiated among the three groups and were used for the investigations of cortical regions.

The correlations between the global cortical diffusion values and global cortical thickness were also tested. The Partial Spearman’s rank correlation analysis revealed that the cortical thickness was significantly correlated with PerpPD (*r* = − 0.715; *p* = 0.000) and ParlPD values (*r* = − 0.664; *p* = 0.000). Correlation analysis to investigate the relationship between the illness duration and global diffusion values revealed a significant positive correlation with PerpPD (*r* = 0.436; *p* = 0.010) and ParlPD values (*r* = 0.469; *p* = 0.005). Finally, the Spearman’s rank correlation showed no significant correlations between head movement and cortical diffusion values.

### Cortical regions

**Fig. 2:**
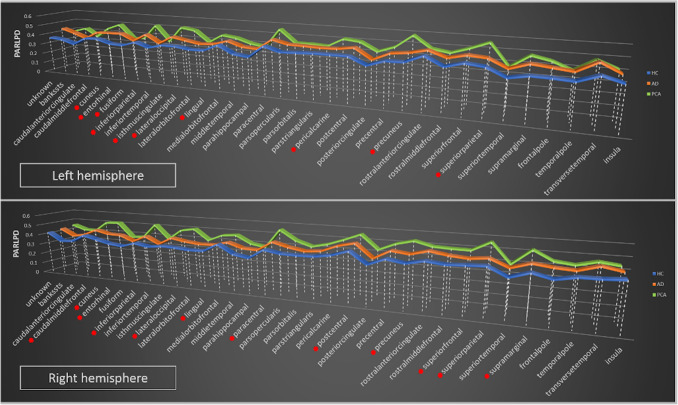
Regional ParlPD differences. Graphs show the group differences in regional ParlPD values. *Remained statistically significant after false discovery rate correction (FDR < 0.05; 210 tests)

Cortical regional comparisons are summarized in Tables 3 and 4 and shown in Figs. 1 and 2 (see also Supplemental file). The GLM analysis revealed multiple significantly different regional PerpPD and ParlPD values in both hemispheres.

**Table 3:** PerpPD regional differences

PerpPD	Left hemisphere					Right hemisphere				
	GLM		Between groups			GLM		Between groups		
	F	*p*	HC vs tAD	HC vs PCA	tAD vs PCA	F	*p*	HC vs tAD	HC vs PCA	tAD vs PCA
Unknown	6.70	0.003*	0.004*	0.094	0.637	1.90	0.160	0.410	0.123	0.669
Bankssts	8.95	0.000*	0.001*	0.002*	0.979	13.46	0.000*	0.001*	0.000*	0.086
Caudalanteriorcingulate	8.94	0.000*	0.001*	0.048	0.571	16.42	0.000*	0.003*	0.000*	0.041
Caudalmiddlefrontal	12.08	0.000*	0.002*	0.000*	0.612	11.79	0.000*	0.007*	0.000*	0.107
Cuneus	13.23	0.000*	0.058	0.000*	0.003*	15.53	0.000*	0.008*	0.000*	0.004*
Entorhinal	9.15	0.000*	0.000*	0.621	0.011*	6.67	0.000*	0.000*	0.075	0.161
Fusiform	32.19	0.000*	0.000*	0.000*	0.001*	21.10	0.000*	0.016*	0.000*	0.000*
Inferiorparietal	16.98	0.000*	0.001*	0.000*	0.019*	22.19	0.000*	0.022*	0.000*	0.000*
Inferiortemporal	4.28	0.019	0.047	0.037	0.927	9.57	0.000*	0.026*	0.000*	0.063
Isthmuscingulate	20.90	0.000*	0.001*	0.000*	0.014*	16.16	0.000*	0.003*	0.000*	0.036
Lateraloccipital	31.41	0.000*	0.022*	0.000*	0.000*	28.27	0.000*	0.026*	0.000*	0.000*
Lateralorbitofrontal	1.33	0.272	0.237	0.857	0.119	2.13	0.129	0.296	0.264	0.969
Lingual	21.25	0.000*	0.011*	0.000*	0.000*	27.58	0.000*	0.018*	0.000*	0.000*
Medialorbitofrontal	0.61	0.547	0.963	0.997	0.949	1.97	0.149	0.157	0.250	0.998
Middletemporal	11.88	0.000*	0.001*	0.002*	0.950	16.68	0.000*	0.002*	0.000*	0.089
Parahippocampal	17.01	0.000*	0.000*	0.000*	0.844	13.40	0.000*	0.002*	0.000*	0.088
Paracentral	3.40	0.041	0.396	0.015*	0.202	10.01	0.000*	0.200	0.000*	0.006*
Parsopercularis	7.40	0.001*	0.001*	0.136	0.284	4.62	0.014	0.235	0.083	0.753
Parsorbitalis	1.29	0.284	0.586	0.394	0.077	2.29	0.111	0.435	0.205	0.807
Parstriangularis	1.91	0.157	0.215	0.675	0.782	2.37	0.103	0.338	0.280	0.958
Pericalcarine	19.80	0.000*	0.062	0.000*	0.036	24.53	0.000*	0.001*	0.000*	0.002*
Postcentral	14.16	0.000*	0.002*	0.000*	0.064	12.29	0.000*	0.016*	0.000*	0.023*
Posteriorcingulate	15.28	0.000*	0.005*	0.000*	0.025*	16.18	0.000*	0.036	0.000*	0.000*
Precentral	11.72	0.000*	0.001*	0.000*	0.527	9.66	0.000*	0.008*	0.000*	0.377
Precuneus	20.36	0.000*	0.006*	0.000*	0.000*	15.48	0.000*	0.024*	0.000*	0.001*
Rostralanteriorcingulate	2.36	0.103	0.071	0.643	0.506	3.67	0.032	0.041	0.289	0.762
Rostralmiddlefrontal	16.23	0.000*	0.000*	0.000*	0.837	10.01	0.000*	0.008*	0.001*	0.479
Superiorfrontal	8.04	0.001*	0.010*	0.006*	0.870	14.06	0.000*	0.011*	0.000*	0.040
Superiorparietal	15.90	0.000*	0.093	0.000*	0.000*	14.51	0.000*	0.172	0.000*	0.000*
Superiortemporal	15.59	0.000*	0.000*	0.000*	0.948	9.32	0.000*	0.005*	0.001*	0.578
Supramarginal	10.62	0.000*	0.001*	0.000*	0.592	15.97	0.000*	0.018*	0.000*	0.001*
Frontalpole	1.83	0.170	0.448	0.354	0.949	4.43	0.017	0.187	0.029	0.539
Temporalpole	0.69	0.504	0.564	0.945	0.821	0.47	0.626	0.999	0.859	0.875
Transversetemporal	21.52	0.000*	0.000*	0.000*	0.949	17.25	0.000*	0.001*	0.005*	0.993
Insula	14.85	0.000*	0.000*	0.014*	0.211	11.16	0.000*	0.001*	0.013*	0.915

The group differences for PerpPD regional measures, investigated using *t* tests. All *p* values remained statistically significant after false discovery rate correction (FDR < 0.05; 210 tests)

*HC* healthy controls, *tAD* typical Alzheimer’s disease, *PCA* posterior cortical atrophy, *GLM* general linear model, *PerpPD* the principal diffusion component perpendicular to the radial minicolumnar direction

*and in bold: Statistically significant difference after false discovery rate correction

**Table 4:** ParlPD regional differences

	Left hemisphere					Right hemisphere				
ParlPD	GLM		Between groups			GLM		Between groups		
	F	*p*	HC vs tAD	HC vs PCA	tAD vs PCA	F	*p*	HC vs tAD	HC vs PCA	tAD vs PCA
Unknown	7.59	0.001*	0.001*	0.618	0.037	1.36	0.266	0.751	0.333	0.709
Bankssts	10.39	0.000*	0.008*	0.000*	0.277	8.69	0.001*	0.144	0.000*	0.032
Caudalanteriorcingulate	1.78	0.178	0.104	0.376	0.867	3.22	0.048	0.078	0.016	0.648
Caudalmiddlefrontal	8.68	0.001*	0.011*	0.001*	0.397	8.21	0.001*	0.194	0.000*	0.017*
Cuneus	12.21	0.000*	0.100	0.000*	0.002*	10.79	0.000*	0.325	0.000*	0.001*
Entorhinal	7.76	0.001*	0.000*	0.995	0.001*	5.39	0.007*	0.001*	0.721	0.003*
Fusiform	2.33	0.107	0.136	0.250	0.992	4.23	0.012*	0.159	0.008*	0.307
Inferiorparietal	17.05	0.000*	0.011*	0.000*	0.002*	22.08	0.000*	0.050	0.000*	0.000*
Inferiortemporal	0.87	0.425	0.329	0.948	0.593	1.35	0.268	0.946	0.267	0.157
Isthmuscingulate	15.45	0.000*	0.012*	0.000*	0.005*	8.87	0.000*	0.068	0.000*	0.105
Lateraloccipital	22.41	0.000*	0.062	0.000*	0.000*	27.83	0.000*	0.125	0.000*	0.000*
Lateralorbitofrontal	3.54	0.036	0.023	0.547	0.356	7.18	0.002*	0.048	0.016*	0.756
Lingual	15.04	0.000*	0.056	0.000*	0.001*	11.11	0.000*	0.199	0.000*	0.002*
Medialorbitofrontal	0.56	0.572	0.870	0.900	1.000	2.81	0.069	0.770	0.235	0.067
Middletemporal	7.45	0.001*	0.006*	0.012*	0.992	5.33	0.008*	0.122	**0.020**	0.573
Parahippocampal	3.25	0.046*	0.030	0.185	0.845	6.30	0.004*	0.006*	0.036	0.942
Paracentral	4.15	0.0021*	0.515	0.006*	0.072	9.77	0.000*	0.233	0.000*	0.007*
Parsopercularis	2.37	0.103	0.055	0.482	0.607	11.30	0.000*	0.189	0.003*	0.138
Parsorbitalis	1.62	0.206	0.361	0.780	0.844	1.51	0.230	0.915	0.479	0.696
Parstriangularis	0.63	0.536	0.367	1.000	0.455	5.75	0.005*	0.569	0.010*	0.092
Pericalcarine	12.00	0.000*	0.392	0.000*	0.004*	9.23	0.000*	0.018*	0.001*	0.434
Postcentral	11.40	0.000*	0.005*	0.000*	0.272	12.96	0.000*	0.037	0.000*	0.009*
Posteriorcingulate	4.416	0.017	0.711	0.027	0.130	4.24	0.020*	0.567	0.007*	0.071
Precentral	9.72	0.000*	0.023	0.000*	0.170	6.89	0.002*	0.053	0.001*	0.221
Precuneus	13.56	0.000*	0.175	0.000*	0.000*	10.97	0.000*	0.129	0.000*	0.003*
Rostralanteriorcingulate	0.73	0.487	0.824	0.959	0.698	3.10	0.053	0.101	0.101	0.971
Rostralmiddlefrontal	11.46	0.000*	0.006*	0.002*	0.739	9.93	0.000*	0.017*	0.000*	0.275
Superiorfrontal	7.20	0.002*	0.062	0.002*	0.242	9.24	0.000*	0.218	0.000*	0.013*
Superiorparietal	10.17	0.000*	0.289	0.000*	0.001*	15.91	0.000*	0.320	0.000*	0.000*
Superiortemporal	6.01	0.004*	0.002*	0.592	0.072	5.88	0.005*	0.010*	0.078	0.876
Supramarginal	15.67	0.000*	0.001*	0.000*	0.115	20.86	0.000*	0.050	0.000*	0.000*
Frontalpole	2.31	0.109	0.355	0.212	0.883	2.86	0.066	0.277	0.183	0.911
Temporalpole	1.78	0.178	0.109	0.983	0.227	0.47	0.624	0.868	0.777	0.972
Transversetemporal	5.99	0.005*	0.004*	0.121	0.595	2.38	0.047	0.041	0.098	0.993
Insula	4.26	0.019	0.020	0.128	0.871	1.83	0.170	0.427	0.275	0.900

The group differences for ParlPD regional measures, investigated using *t* tests. All *p* values remained statistically significant after false discovery rate correction (FDR < 0.05; 210 tests)

*HC* healthy controls, *tAD* typical Alzheimer’s disease, *PCA* posterior cortical atrophy, *GLM* general linear model, *ParlPD* the principal diffusion component parallel with the radial minicolumnar direction

* and in bold: Statistically significant difference after false discovery rate correction

As expected, the between groups comparisons (Tables 3, 4) revealed that both patient groups exhibited higher PerpPD and ParlPD values compared to the HC group in a large number of frontal, temporal, parietal and occipital cortical regions. Comparing directly the two patient groups, the PCA group revealed higher PerpPD and ParlPD values in a large number of parieto-occipital regions (e.g., inferior parietal, lateral occipital, lingual, precuneus, superior parietal and supramarginal cortex), and the tAD group had higher PerpPD in the left entorhinal cortex and higher ParlPD in bilateral entorhinal cortex (FDR < 0.05; 210 tests).

### Receiver operating characteristic (ROC) curves analysis

#### Global cortical

The AUC of global diffusion and structural measures are summarized in Table 5. The AngleR was the best measure to discriminate HC from all patients grouped together (AUC = 0.902) and to discriminate HC from tAD (AUC = 0.922), while PerpPD was the best measure to discriminate HC from PCA (AUC = 0.961). Finally, the best global measure to differentiate the two patient groups was ParlPD (AUC = 0.771).

**Table 5:** Global cortical diffusion and volumetrics AUC

Measures	HC vs all patients AUC	HC vs tAD AUC	HC vs PCA AUC	tAD vs PCA AUC
FA	0.629	0.625	0.635	0.528
MD	0.830	0.812	0.858	0.646
AngleR	***0.902***	***0.922***	0.873	0.559
PerpPD	0.889	0.862	***0.961***	0.731
ParlPD	0.836	0.777	0.927	***0.771***
Cortical GM_fr	0.874	0.861	0.911	0.645
Cortical Thickness	0.811	0.781	0.870	0.658
Hipp_fr	0.838	0.856	0.798	0.688

This table resumed the AUC for each measure comparing the groups with each other

*AUC* area under the curve, *HC* healthy controls, *tAD* typical Alzheimer’s disease, *PCA* posterior cortical atrophy, *FA* fractional anisotropy, *MD* mean diffusivity, *AngleR* angle between the radial minicolumnar direction and the principal diffusion direction, *PerpPD* the principal diffusion component perpendicular to the radial minicolumnar direction, *ParlPD* the principal diffusion component parallel with the radial minicolumnar direction, *Hipp_fr* hippocampal fraction. In bold the highest AUC for each comparison

#### Cortical regions

Tables 6 and 7 show, respectively, the results of ROC analysis performed on PerpPD and ParlPD regional values. Both measures were able to discriminate HC, tAD and PCA with an accuracy ranging from good to excellent across a large number of regions (AUC from 0.800 to 0.945). Thus, the entorhinal, fusiform, middle temporal, parahippocampal, superior temporal, transverse temporal and insula cortex exhibited the most separation between HC and tAD; while fusiform, inferior parietal, lateral occipital, lingual, postcentral, precuneus, superior parietal and supramarginal cortex exhibited the best separation between HC and PCA.

**Table 6:** Regional PerpPD AUC

PerpPD left hemisphere	HC vs tAD	HC vs PCA	tAD vs PCA	PerpPD right hemisphere	HC vs tAD	HC vs PCA	tAD vs PCA
Unknown	0.783	0.733	0.562	Unknown	0.630	0.642	0.533
Bankssts	0.755	**0.842**	0.557	Bankssts	**0.808**	**0.873**	0.600
Caudalanteriorcingulate	**0.824**	0.752	0.571	Caudalanteriorcingulate	**0.800**	**0.903**	0.629
Caudalmiddlefrontal	0.765	**0.876**	0.597	Caudalmiddlefrontal	**0.806**	**0.812**	0.620
Cuneus	0.735	**0.855**	0.670	Cuneus	**0.782**	**0.871**	0.704
Entorhinal	**0.862**	0.588	**0.801**	Entorhinal	**0.831**	0.679	0.662
Fusiform	**0.858**	**0.933**	0.765	Fusiform	**0.852**	**0.897**	**0.802**
Inferiorparietal	**0.806**	**0.909**	0.695	Inferiorparietal	0.761	**0.933**	0.759
Inferiortemporal	0.652	0.712	0.551	Inferiortemporal	0.762	**0.806**	0.591
Isthmuscingulate	**0.801**	**0.918**	0.681	Isthmuscingulate	**0.852**	**0.888**	0.629
Lateraloccipital	0.763	**0.912**	**0.817**	Lateraloccipital	**0.808**	**0.906**	**0.811**
Lateralorbitofrontal	0.617	0.552	0.575	Lateralorbitofrontal	0.640	0.645	0.522
Lingual	0.789	**0.909**	**0.802**	Lingual	**0.801**	**0.936**	**0.856**
Medialorbitofrontal	0.502	0.585	0.606	Medialorbitofrontal	0.668	0.673	0.611
Middletemporal	**0.804**	**0.882**	0.600	Middletemporal	**0.818**	**0.906**	0.664
Parahippocampal	**0.840**	**0.897**	0.562	Parahippocampal	**0.832**	**0.879**	0.559
Paracentral	0.656	0.706	0.559	Paracentral	0.660	**0.842**	0.693
Parsopercularis	0.765	**0.806**	0.587	Parsopercularis	0.664	0.709	0.574
Parsorbitalis	0.565	0.673	0.767	Parsorbitalis	0.579	0.664	0.594
Parstriangularis	0.628	0.645	0.519	Parstriangularis	0.654	0.682	0.586
Pericalcarine	0.719	**0.891**	0.754	Pericalcarine	**0.842**	**0.909**	0.704
Postcentral	0.769	**0.909**	0.676	Postcentral	0.751	**0.830**	0.684
Posteriorcingulate	0.749	**0.927**	0.685	Posteriorcingulate	0.745	**0.876**	0.701
Precentral	0.781	**0.864**	0.559	Precentral	0.759	0.774	0.603
Precuneus	**0.802**	**0.897**	**0.802**	Precuneus	**0.803**	**0.872**	0.796
Rostralanteriorcingulate	0.660	0.661	0.612	Rostralanteriorcingulate	0.741	0.673	0.626
Rostralmiddlefrontal	**0.808**	**0.918**	0.530	Rostralmiddlefrontal	0.755	**0.855**	0.580
Superiorfrontal	0.743	0.796	0.548	Superiorfrontal	0.767	**0.882**	0.658
Superiorparietal	0.711	**0.891**	**0.806**	Superiorparietal	0.684	**0.845**	**0.842**
Superiortemporal	**0.814**	**0.897**	0.597	Superiortemporal	**0.834**	**0.848**	0.551
Supramarginal	**0.815**	**0.877**	0.559	Supramarginal	0.763	**0.870**	0.687
Frontalpole	0.619	0.694	0.614	Frontalpole	0.698	0.727	0.525
Temporalpole	0.542	0.582	0.545	Temporalpole	0.589	0.652	0.588
Transversetemporal	**0.891**	**0.864**	0.657	Transversetemporal	**0.830**	**0.809**	0.659
Insula	**0.858**	**0.876**	0.636	Insula	**0.872**	**0.800**	0.665

This table shows the AUC for each regional PerpPD values comparing the three groups with each other. In bold AUC > 0.80. HC = healthy controls, tAD = typical Alzheimer’s disease; PCA = posterior cortical atrophy; PerpPD = the principal diffusion component perpendicular to the radial minicolumnar direction

**Table 7:** Regional ParlPD AUC

ParlPD Left hemisphere	HC vs tAD	HC vs PCA	tAD vs PCA	ParlPD Right hemisphere	HC vs tAD	HC vs PCA	tAD vs PCA
Unknown	**0.810**	0.709	0.600	Unknown	0.615	0.721	0.655
Bankssts	0.798	0.797	0.574	Bankssts	0.725	0.773	0.614
Caudalanteriorcingulate	0.656	0.636	0.639	Caudalanteriorcingulate	0.672	0.739	0.575
Caudalmiddlefrontal	0.727	**0.858**	0.617	Caudalmiddlefrontal	0.668	**0.806**	0.658
Cuneus	0.761	**0.827**	0.632	Cuneus	0.680	**0.831**	0.704
Entorhinal	**0.815**	0.528	**0.801**	Entorhinal	**0.807**	0.558	0.786
Fusiform	0.692	0.633	0.519	Fusiform	0.708	0.697	0.582
Inferiorparietal	0.702	**0.945**	0.704	Inferiorparietal	0.789	**0.897**	**0.811**
Inferiortemporal	0.589	0.515	0.584	Inferiortemporal	0.514	0.561	0.533
Isthmuscingulate	0.777	**0.852**	0.710	Isthmuscingulate	0.700	0.764	0.623
Lateraloccipital	0.783	**0.901**	**0.817**	Lateraloccipital	0.769	**0.903**	**0.826**
Lateralorbitofrontal	0.678	0.712	0.622	Lateralorbitofrontal	0.735	0.761	0.629
Lingual	0.672	**0.918**	0.751	Lingual	0.700	**0.818**	0.696
Medialorbitofrontal	0.522	0.588	0.597	Medialorbitofrontal	0.565	0.606	0.690
Middletemporal	0.767	0.788	0.525	Middletemporal	0.729	0.752	0.583
Parahippocampal	0.708	0.579	0.616	Parahippocampal	0.796	0.652	0.586
Paracentral	0.611	0.742	0.600	Paracentral	0.644	**0.815**	0.681
Parsopercularis	0.644	0.694	0.574	Parsopercularis	0.640	**0.833**	0.707
Parsorbitalis	0.686	0.585	0.588	Parsorbitalis	0.502	0.597	0.620
Parstriangularis	0.593	0.567	0.594	Parstriangularis	0.609	0.779	0.652
Pericalcarine	0.630	**0.830**	0.788	Pericalcarine	0.763	**0.812**	0.641
Postcentral	0.779	**0.873**	0.655	Postcentral	0.713	**0.870**	0.719
Posteriorcingulate	0.585	0.770	0.675	Posteriorcingulate	0.605	0.770	0.658
Precentral	0.711	**0.879**	0.652	Precentral	0.709	0.779	0.571
Precuneus	0.585	**0.855**	**0.803**	Precuneus	0.678	**0.830**	0.713
Rostralanteriorcingulate	0.694	0.515	0.513	Rostralanteriorcingulate	0.668	0.676	0.545
Rostralmiddlefrontal	0.747	**0.894**	0.617	Rostralmiddlefrontal	0.741	**0.812**	0.614
Superiorfrontal	0.690	**0.833**	0.638	Superiorfrontal	0.662	**0.847**	0.693
Superiorparietal	0.686	**0.836**	0.738	Superiorparietal	0.658	**0.848**	**0.814**
Superiortemporal	0.725	0.727	0.577	Superiortemporal	0.765	0.730	0.667
Supramarginal	**0.826**	**0.915**	0.613	Supramarginal	0.741	**0.936**	**0.825**
Frontalpole	0.615	0.712	0.583	Frontalpole	0.654	0.736	0.571
Temporalpole	0.593	0.609	0.521	Temporalpole	0.583	0.648	0.545
Transversetemporal	0.773	0.676	0.559	Transversetemporal	0.674	0.767	0.565
Insula	0.708	0.773	0.576	Insula	0.625	0.624	0.507

The AUC for each regional ParlPD values comparing the three groups with each other. In bold AUC > 0.80

*HC* healthy controls, *tAD* typical Alzheimer’s disease, *PCA* posterior cortical atrophy, *ParlPD* the principal diffusion component parallel with the radial minicolumnar direction

To discriminate the two patient groups, the cortical regions with higher AUC were entorhinal, inferior parietal, lateral occipital, lingual, precuneus, superior parietal and supramarginal cortex.

## Conclusion

Differences in cortical diffusivity measures between PCA patients, healthy controls, and patients with tAD were investigated in this study. The analysis assessed if there were between-group differences in global cortical diffusivity and regional cortical diffusivity and explored their discrimination power using ROC curves.

### Global cortical diffusivity

The results showed significant differences between all groups for the PerpPD and ParlPD global values, with the PCA group presenting the higher values. Based on previous studies [21, 33, 34], these measures and other, related, cortical diffusivity values may act as surrogate measures of cytoarchitectural features such as minicolumn structure and may also be sensitive to changes in organization related to neuropathology including pathological protein accumulation.

Neurodegeneration in Alzheimer’s disease (and other dementias) proceeds insidiously and gradually from synaptic damage to dendrite loss, to cell loss and then to large scale atrophy. With respect to cytoarchitectural organisation there is shrinkage of horizontal cortical layers associated with cortical thinning and there is shrinkage and disruption of vertical minicolumn organisation. One concept in previous work [25] is that the minicolumn width shrinks initially due to loss of neuropil between cells, and then, in severe AD, there is minicolumn breakdown due to tangle accumulation and cell loss. It is possible that these phases of progression could be associated with differences in the characteristics of diffusion in the cortex due to changes in microscopic geometry of cytoarchitecture [35]. Furthermore, it appears that the different patterns of neurodegeneration between PCA and AD may be reflected in some measures of cortical diffusivity more than others, in this case in differences in PerpPD and ParlPD.

The global grey matter fraction and cortical surface area were reduced in both patient groups compared to the healthy control group, with no significant differences between tAD and PCA groups. However, the PCA group also had a reduced cortical thickness compared to the tAD group. Therefore, both of the contributing components of cortical volume (surface area and thickness) were affected in PCA and to some extent worse that in tAD. This is consistent with more severe changes that may be due to the longer illness duration of PCA. Illness duration was also correlated with PerpPD and ParlPD values across this dataset. The additional finding that cortical thickness was negatively correlated with global cortical PerpPD and ParlPD suggests there may be a meaningful coincidence of longer duration of illness and more severe cortical thickness and cortical diffusivity damage in PCA.

### Regional cortical diffusivity

**Fig. 3:**
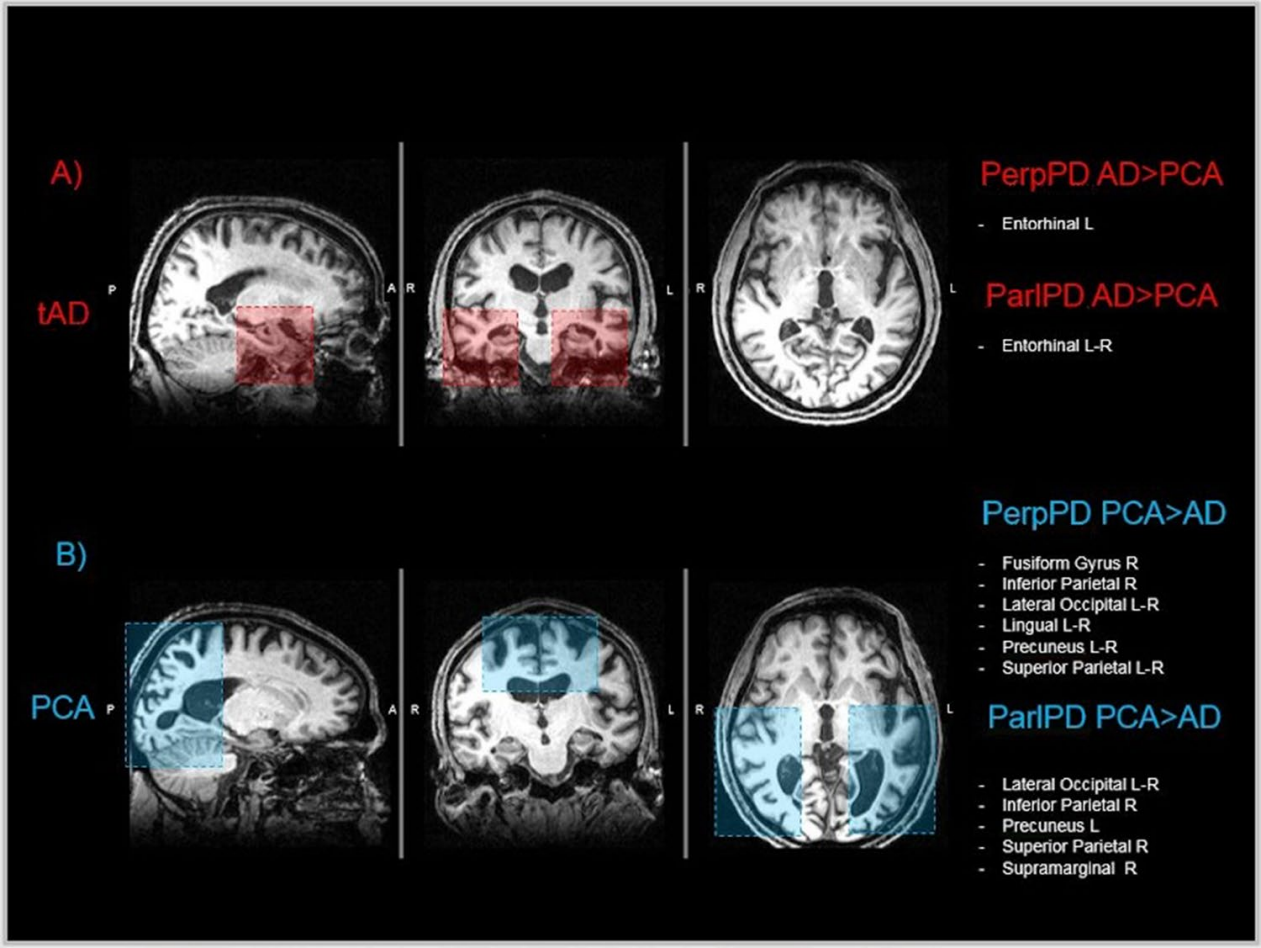
Cortical pattern**.** The figure summarizes the main regional cortical PerpPD and ParlPD pattern for each patient group. As previously described, the tAD group, compared with the tAD group, showed an increased cortical PerpPD and ParlPD pattern mainly focused on temporal regions. Conversely, the PCA group showed a cortical PerpPD pattern, mainly focused on parietal-occipital regions

Consistent with previous works using other forms of assessment [13, 17, 36], the regional comparisons revealed that the PCA and tAD subgroups showed two different patterns of cortical changes (Fig. 3). Compared with healthy controls, PCA patients showed significantly higher values of PerpPD and ParlPD in bilateral parietal and occipital regions (e.g., inferior parietal, lateral occipital, lingual, superior parietal, supramarginal, precuneus). In contrast, tAD patients demonstrated significantly higher PerpPD and ParlPD values, compared with controls, in temporal regions (e.g., entorhinal, middle temporal, parahippocampal, superior temporal, transverse temporal, insula). Comparing the two patient groups directly, tAD patients had higher PerpPD in left entorhinal cortex and higher ParlPD values in bilateral entorhinal cortex than PCA patients. As is well known, the entorhinal region is the earliest one affected in tAD neurodegeneration [13, 37], contributing to the memory symptoms reported in tAD.

Compared with tAD patients, PCA patients had higher PerpPD and ParlPD values mainly in some posterior cortical regions, usually related to visuospatial and visuoperceptual abilities, encompassing bilateral fusiform, bilateral cuneus, bilateral inferior parietal, bilateral lateral occipital, bilateral lingual, bilateral precuneus, bilateral superior parietal, right paracentral and right supramarginal cortex. As shown in other studies [38, 39], these regions are involved in visual perception tasks and their damage can cause the deficits that characterize the clinical presentation of PCA.

This different pattern of cortical changes seems to be confirmed also by volumetric comparisons in which both patient groups showed a lower bilateral hippocampal fraction compared to healthy controls but also a lower bilateral hippocampal fraction in tAD compared with PCA.

Concerning the discrimination power of these cortical diffusivity measures, this study demonstrated that the global measures enabled differentiation with excellent accuracy between HC and all patients grouped together (AngleR AUC = 0.902), between HC and tAD (AngleR = 0.922) and between HC and PCA (PerpPD AUC = 0.961). However, to differentiate between the two patient groups the global cortical volumetric and diffusion measures were of limited use [ParlPD demonstrated the best AUC (0.771)].

According to previous studies [40–42], tAD and PCA could have the same underlying neuropathology, but a different cortical distribution, therefore, a global cortical measure may not be suitable, or sensitive enough, to discriminate between the two dementia groups.

The regional cortical values were found to differentiate tAD and PCA patients with a higher discrimination accuracy than global cortical values. The best AUC was shown by PerpPD right lingual cortex (AUC = 0.856), PerpPD right superior parietal cortex (AUC = 0.842) and ParlPD right lateral occipital cortex (AUC = 0.826). These regions are involved in visual-spatial tasks and represent key regions in the PCA cortical signature.

These findings further confirm an association between the novel cortical diffusivity measurements and the cortical region size changes observed in both PCA and tAD patients, suggesting a potential role for DTI measures to enhance assessment of cortical changes in dementia.

The novel cortical diffusion measures described could have several applications. Further studies will enable a greater understanding of the underlying pathological cortical changes in the brain caused by various neurodegenerative diseases. Novel cortical diffusion measures may prove to be useful for investigating the cytoarchitectural organization changes among dementia types and could be an effective tool to aid differential diagnosis.

### Study limitations

Despite the novel findings described above, there are several limitations to the current study. First, the study included a relatively small number of subjects because of the rarity of PCA, therefore these findings should be replicated with a larger number of subjects. Additionally, no neuropsychological comparisons between groups were possible because the two patient groups were originally enrolled in two different projects using different batteries of neuropsychological tools. The patients were diagnosed and classified as tAD and PCA on the basis of clinical presentation, so we cannot be certain about the neuropathology underlying the symptoms and the possibility that some patients may have coexistent, additional neuropathologies cannot be excluded. Finally, the cortical diffusivity measures presented here were not directly shown in this study to measure minicolumn organization or any specific cytoarchitectural component and may instead represent a surrogate of the combination of cortical changes occurring during neurodegeneration, most likely including minicolumn breakdown and other processes.

In conclusion, different diffusion values were found, predominantly in the posterior cortical and temporal regions, between PCA and tAD subjects. These novel diffusion values showed excellent discrimination power to differentiate between tAD and PCA. This study provides insights into the role that cortical diffusivity could play as a potential method for investigating the microstructural changes in diverse neuropathological conditions. Further studies with ex-vivo cohorts and larger in-vivo cohorts will be necessary to generalize these findings.

## Electronic supplementary material

Below is the link to the electronic supplementary material.

Supplementary file1 (DOCX 219 kb)

## Data Availability

The data that support the findings of this study are available from the corresponding author upon reasonable request.
