# Supplementary material for: Cortical diffusivity investigation in posterior cortical atrophy and typical Alzheimer’s disease
Source: J Neurol. 2020 Aug 8;268(1):227–39. doi: 10.1007/s00415-020-10109-w (PMC7815619; doi:10.1007/s00415-020-10109-w)
Supplement: Supplementary file 1 — Supplementary file1 (DOCX 219 kb) [file 415_2020_10109_MOESM1_ESM.docx]

**Supplemental**

**Figure 1 Bilateral regional PerpPD**


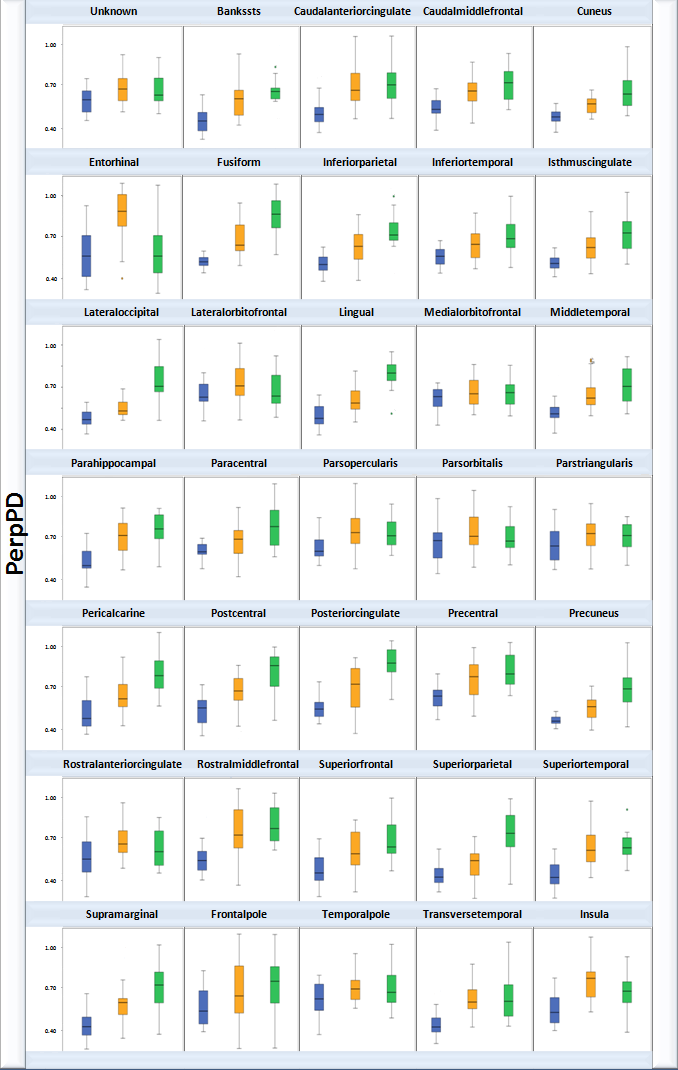


Figure 1: For each region the bilateral value was calculated by averaging the left and right values. The box plots show the regional bilateral PerpPD for each group. (Controls = blue, tAD= orange, PCA= green).

**Figure 2 Bilateral regional ParlPD**

**
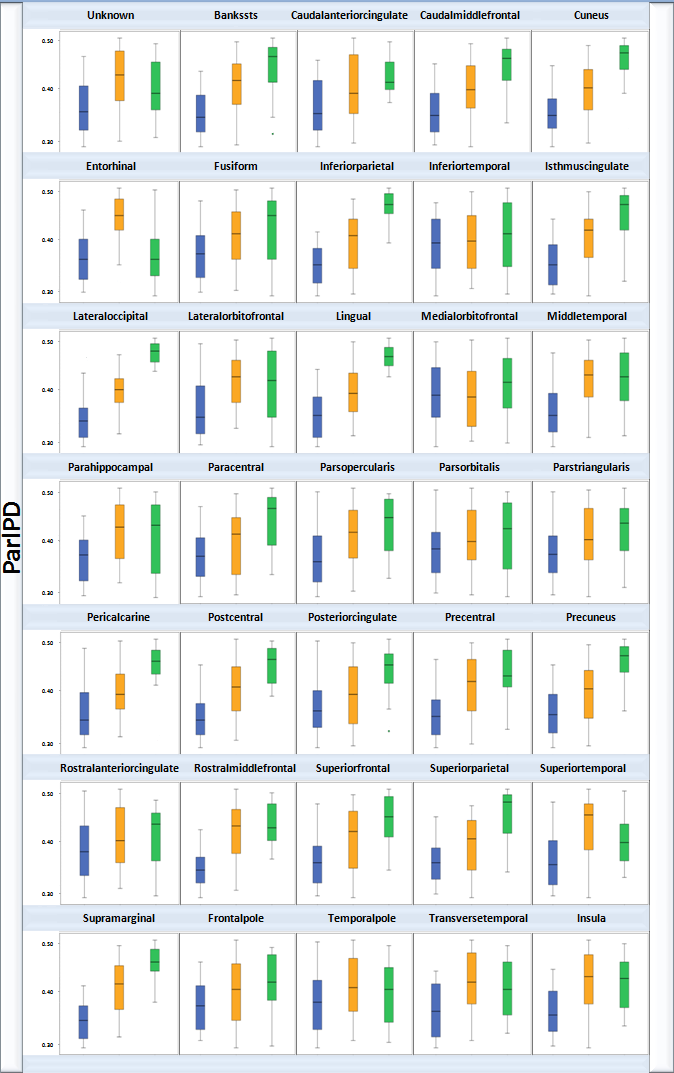
**

Figure 2: For each region the bilateral value was calculated by averaging the left and right values. The box plots show the regional bilateral ParlPD for each group. (Controls = blue, tAD= orange, PCA= green).
